# Recurrent loss of heterozygosity correlates with clinical outcome in pancreatic neuroendocrine cancer

**DOI:** 10.1038/s41525-018-0058-3

**Published:** 2018-07-20

**Authors:** Ben Lawrence, Cherie Blenkiron, Kate Parker, Peter Tsai, Sandra Fitzgerald, Paula Shields, Tamsin Robb, Mee Ling Yeong, Nicole Kramer, Sarah James, Mik Black, Vicky Fan, Nooriyah Poonawala, Patrick Yap, Esther Coats, Braden Woodhouse, Reena Ramsaroop, Masato Yozu, Bridget Robinson, Kimiora Henare, Jonathan Koea, Peter Johnston, Richard Carroll, Saxon Connor, Helen Morrin, Marianne Elston, Christopher Jackson, Papaarangi Reid, John Windsor, Andrew MacCormick, Richard Babor, Adam Bartlett, Dragan Damianovich, Nicholas Knowlton, Sean Grimmond, Michael Findlay, Cristin Print

**Affiliations:** 1 Discipline of Oncology, Faculty of Medicine and Health Sciences, University of Auckland, Auckland, New Zealand; 2 Maurice Wilkins Centre hosted by the University of Auckland, Auckland, New Zealand; 3 Department of Molecular Medicine and Pathology, School of Medical Sciences, Faculty of Medicine and Health Sciences, University of Auckland, Auckland, New Zealand; 4 Anatomic Pathology Services, Auckland, New Zealand; 5 LabPlus, Auckland City Hospital, Auckland, New Zealand; 6 Department of Biochemistry, University of Otago, Dunedin, New Zealand; 7 Bioinformatics Institute, University of Auckland, Auckland, New Zealand; 8 Genetic Health Service New Zealand (Northern Hub), Auckland, New Zealand; 9 Waitemata District Health Board, Auckland, New Zealand; 10 Histopathology Department, Middlemore Hospital, Auckland, New Zealand; 11 Canterbury District Health Board, Christchurch, New Zealand; 12 Auckland Cancer Society Research Centre, Faculty of Medical and Health Sciences, The University of Auckland, Auckland, New Zealand; 13 Upper Gastrointestinal Unit, Department of Surgery, North Shore Hospital, Takapuna, Auckland, New Zealand; 14 Department of Surgery, Auckland District Health Board, Auckland, New Zealand; 15 Endocrine, Diabetes and Research Centre, Wellington Regional Hospital, Wellington, New Zealand; 16 Department of Pathology, University of Otago Christchurch, Christchurch, New Zealand; 17 Waikato Clinical Campus, University of Auckland Department of Medicine, Auckland, New Zealand; 18 Department of Medicine, Dunedin School of Medicine, University of Otago, Dunedin, New Zealand; 19 Te Kupenga Hauora Māori, Faculty of Medical and Health Sciences, University of Auckland, Auckland, New Zealand; 20 Department of Surgery, University of Auckland, Auckland, New Zealand; 21 Department of Surgery, Counties Manukau District Health Board, Auckland, New Zealand; 22 Department of Medical Oncology, Auckland City Hospital, Auckland, New Zealand; 23 University of Melbourne Centre for Cancer Research, University of Melbourne, Melbourne, Victoria Australia

## Abstract

Pancreatic neuroendocrine tumors (pNETs) are uncommon cancers arising from pancreatic islet cells. Here we report the analysis of gene mutation, copy number, and RNA expression of 57 sporadic well-differentiated pNETs. pNET genomes are dominated by aneuploidy, leading to concordant changes in RNA expression at the level of whole chromosomes and chromosome segments. We observed two distinct patterns of somatic pNET aneuploidy that are associated with tumor pathology and patient prognosis. Approximately 26% of the patients in this series had pNETs with genomes characterized by recurrent loss of heterozygosity (LoH) of 10 specific chromosomes, accompanied by bi-allelic *MEN1* inactivation and generally poor clinical outcome. Another ~40% of patients had pNETs that lacked this recurrent LoH pattern but had chromosome 11 LoH, bi-allelic *MEN1* inactivation, and universally good clinical outcome. The somatic aneuploidy allowed pathogenic germline variants (e.g., *ATM*) to be expressed unopposed, with RNA expression patterns showing inactivation of downstream tumor suppressor pathways. No prognostic associations were found with tumor morphology, single gene mutation, or expression of RNAs reflecting the activity of immune, differentiation, proliferative or tumor suppressor pathways. In pNETs, single gene mutations appear to be less important than aneuploidy, with *MEN1* the only statistically significant recurrently mutated driver gene. In addition, only one pNET in the series had clearly actionable single nucleotide variants (SNVs) (in *PTEN* and *FLCN*) confirmed by corroborating RNA expression changes. The two clinically relevant patterns of LoH described here define a novel oncogenic mechanism and a plausible route to genomic precision oncology for this tumor type.

## Cancer: Frequent chromosome loss in rare pancreatic tumors

The loss of entire chromosomes seems to be a fundamental driver of tumors arising from the hormone-producing cells of the pancreas. A team led by Cristin Print and Ben Lawrence from the University of Auckland, New Zealand, performed genomic and pathological analysis of 57 pancreatic neuroendocrine tumors, a rare form of cancer caused by the abnormal growth of hormone-producing islet cells within the pancreas. The researchers observed two distinct patterns of chromosome loss, with 26% of the samples missing one copy of 10 specific chromosomes and another 40% lacking a copy of chromosome 11. In both groups, the abnormal chromosome count prompts abnormal gene activity patterns, with recessive mutations unleashed and expressed unopposed. Single gene mutations seem to play only a minor role, suggesting that single gene-targeted drugs will provide little benefit in this disease setting, with more nuanced approaches required.

## Introduction

Pancreatic neuroendocrine tumors (pNETs) are clinically heterogeneous tumors derived from neuroendocrine cells of pancreatic islets, which follow a variable clinical course, but are fatal in 60% of patients within 5 years.^1^ Effective systemic treatments are emerging including chemotherapies,^2^ radionuclide therapies,^3^ and therapies that target specific molecular changes in tumor cells (e.g., sunitinib,^4^ everolimus^5^). Despite recent recognition of profound biological heterogeneity between pNETs, therapeutic decisions are currently made without knowledge of the biological drivers of each individual pNET, underlining the potential for genomic understanding of these tumors to improve outcomes for patients.

Numerous genomic changes have been observed in well-differentiated pNETs, including telomeric dysregulation,^6^ copy number (CN) changes,^7^ changes in RNA expression that indicate mTOR pathway activation,^8^ germline *MEN1* or *MUTYH* inactivation,^9^changes in methylation,^10^ and changes to the sequence, methylation, and expression of genes encoding epigenetic modifiers.^11^ Specific mutations in tumor suppressor genes are now accepted as drivers of pNET tumorigenesis (e.g., *MEN1*, *DAXX*, *ATRX*, *VHL*, *YY1*, and mechanistic target of rapamycin (mTOR) pathway genes^9,12,13^). The recognition of such a range of genomic changes in pNETs suggests that each tumor might require multimodal genomic analysis to accurately guide therapeutic choice.

While genomic-enabled oncology has been somewhat successful in many tumor types (e.g., lung,^14,15^ colon,^16^ and ovarian^17^ carcinoma), this is not the case in pNETs. Analyses of tumor tissue to search for predictive biomarkers in pNETs are rare and limited to few markers. For example, although the targets of sunitinib are known (e.g., PDGFR-α, β, VEGFR-1, 2, 3, FLT3, RET, KIT), the presence of mutations or changes in target expression have been minimally described.^18^ Response to everolimus, an mTOR inhibitor, has been correlated with mTOR pathway protein expression,^19^ but the presence of pathway member gene mutation and RNA expression changes appear in a smaller proportion of patients than expected from clinical trials, and awaits elucidation. Similarly, successful treatment with the alkylating chemotherapy temozolomide occurs in pNETs with low MGMT protein expression in some but not all studies,^20–22^ with MGMT promoter hypermethylation a possible cause.^23^ Despite these correlations suggesting a relationship between response and tumor biology, the relationships are imperfect, and no predictive biomarker is in current use in the clinic. A deeper multimodal description of pNETs is needed to find and understand the biological targets that these agents work on.

Therefore, we undertook pathological examination and deep multimodal genomic analysis of a group of clinically homogenous well-differentiated sporadic pNETs. Our results show that pNETs are dominated by aneuploidy along with *MEN1* gene mutation and that the extensive loss of heterozygosity (LoH) seen in pNETs is linked to dysregulation of RNA expression on the scale of whole chromosomes. Distinct patterns of recurrent chromosome-level aneuploidy relate to clinical outcome and could inform clinical care.

## Results

**Fig. 1:**
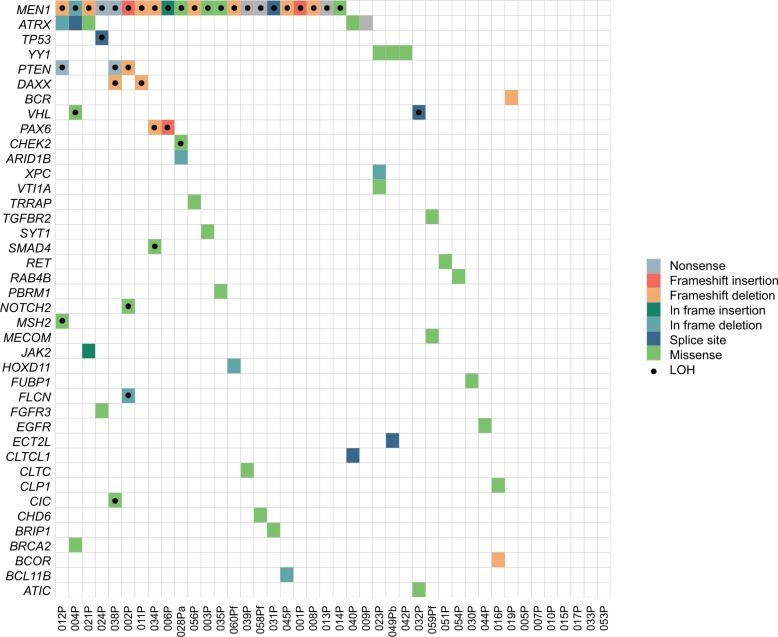
The mutational landscape of pNETs. Coding region somatic non-synonymous SNVs/indels, large deletions, and intronic mutations within 2 bp of splice sites with any putative functional significance (see Methods) are shown. Tumors are indicated in columns and genes in rows. Colored squares indicate mutation type, with dots indicating that loss of the remaining wild-type allele (LoH) could be confirmed for the locus through changes in both allele frequency of germline heterozygous SNPs and normalized relative regional sequence depth in tumor vs. normal samples. In some tumors, there were no detectable mutations in the 637 genes covered by the targeted sequencing panel

**Fig. 2:**
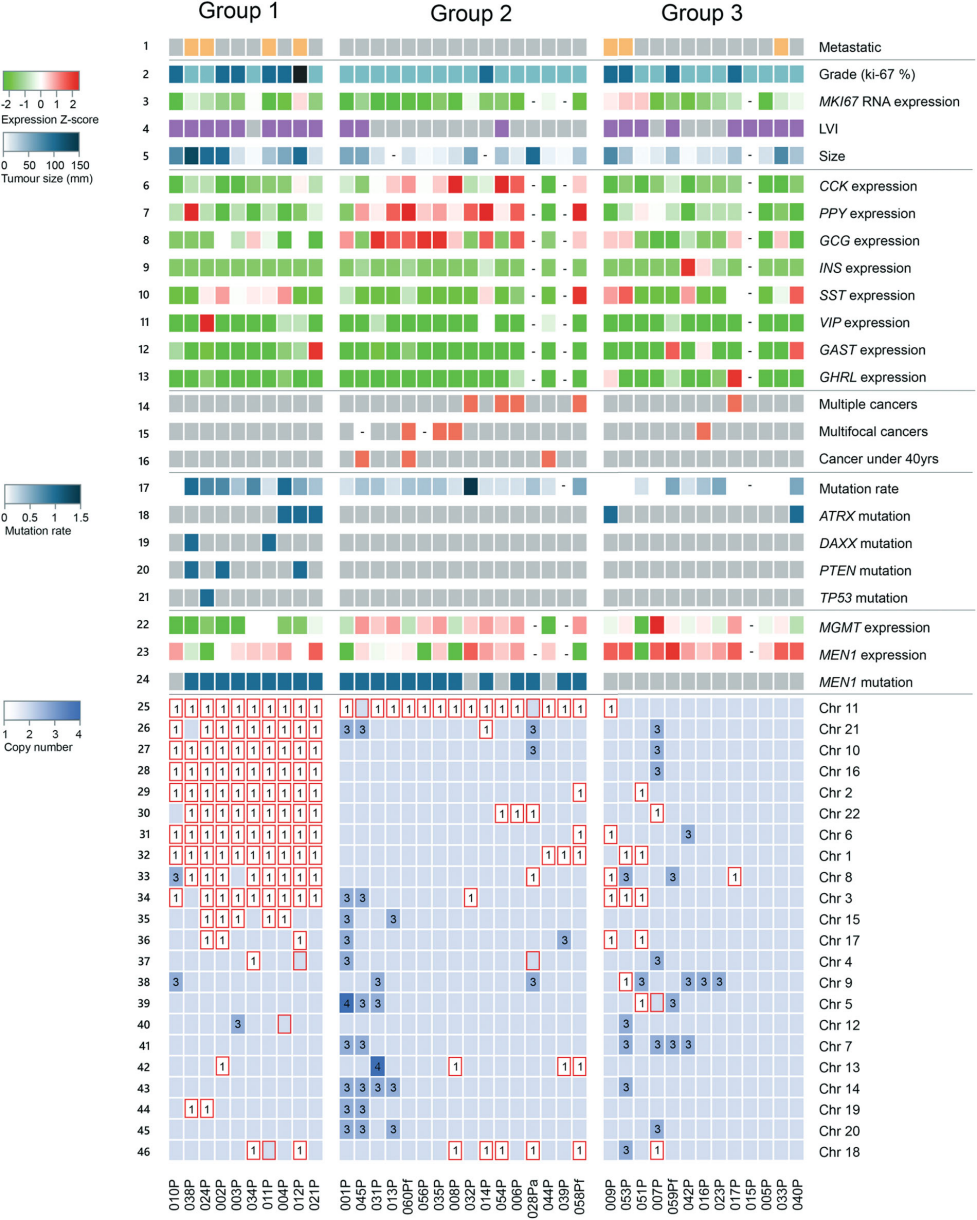
The genomic landscape of pNETs is dominated by aneuploidy. Tumors are shown in columns and genomic and pathological features in rows. Row 1: metastatic tumors are shown in orange. Row 2: Ki67 ≤2% (defined here as grade 1) is shown in light blue, Ki67 3–20% (defined as grade 2) in dark blue, and Ki67 >20% (defined as grade 3) in black. Row 3: *MKI67* RNA expression *Z*-score across tumors (green–red color key to left). Dashes indicate that no expression data were available for specific tumors. Row 4 shows the histological identification of lymphovascular invasion (LVI) in purple, tumors without LVI are colored gray. Row 5 shows tumor size (diameter in mm) on a white–blue scale (white–blue color key to left). Rows 6–13 show expression *Z*-scores across tumors of the following RNAs (green–red color key to left of row 3): *CCK*, *PPY*, *GCG, INS, SST, VIP, GAST*, and *GHRL*. Rows 14–16 show multiple cancers of any type in the same individual, multifocal pNETs, and pNETs arising at under 40 years of age, respectively, indicated by red boxes. Row 17 shows the number of functionally significant exonic mutations on a white–blue scale (white–blue color key to left). In rows 18–21, blue squares indicate somatic mutations in the four listed genes. Rows 22 and 23 show the expression of *MGMT* and *MEN1* mRNA (*Z*-scores, green–red color key to left of row 3). Row 24: Somatic mutations in *MEN1* are shown in blue. In rows 25–46, coloring of blocks indicates the dominant inferred CN for each autosome in each tumor based on combined information from: ADTEx analysis, relative somatic read counts at germline heterozygous positions and normalized read counts in 3 kb tiles across the genome. LoH (irrespective of CN) is indicated by red boxes. Unmarked blue boxes indicate an inferred chromosome CN of 2 and numerals indicate CN when CN ≠ 2

We analyzed a near-sequential series of 57 sporadic pNETs collected from 53 New Zealand patients along with matched normal tissues. For overall study make-up, see Supplementary Fig. S1; key population characteristics are described in Supplementary Table S1, and individual patient characteristics are described in Supplementary Table S2. Cases selected had a clinical and pathological diagnosis of well-differentiated pNET, expressed at least one of the three neuroendocrine immunohistochemical protein markers (chromogranin A, synaptophysin, or CD56), and were surgically resectable at initial diagnosis. Genomic DNA was analyzed from 47 tumors of 43 patients (including 42 primary tumors) using deep hybridization capture sequencing of 637 genes (578 genes previously associated with cancer, plus an additional 59 genes with published or predicted significance for NETs; Supplementary Table S3). In the 42 primary pNETs analyzed, only a small number of somatic single nucleotide variants (SNVs) and indels with putative functional significance were identified (Fig. 1; Supplementary Table S5). However, further analysis of these sequence data identified substantial CN gains and losses, in some cases associated with LoH of large regions of the tumor genome (Fig. 2). RNA expression was then analyzed from 55 tumors of 52 patients (50 primary tumors) using Affymetrix microarrays, informing the interpretation of these somatic mutations and CN DNA changes.

Additional total RNA and mRNA sequence analysis, methylation microarray analysis, and low coverage whole-genome sequencing (WGS) was undertaken for the first 12 tumors in this series for which fresh frozen tissue was available (summarized in Supplementary Table S4). The WGS confirmed the CN changes revealed by the targeted sequencing. In addition, non-negative Matrix Factorization (NMF) mutational signature analysis of the pooled WGS data from these 12 pNETs revealed a putative G:C > T:A signature (Supplementary Fig. S2a-b). Although sequence depth was insufficient to analyze the 12 tumors individually, a mutational signature similar to that found in the pooled tumors has recently been identified in pNETs.^9^

### Aneuploidy defines the molecular landscape of pNETs and alters gene expression

**Fig. 3:**
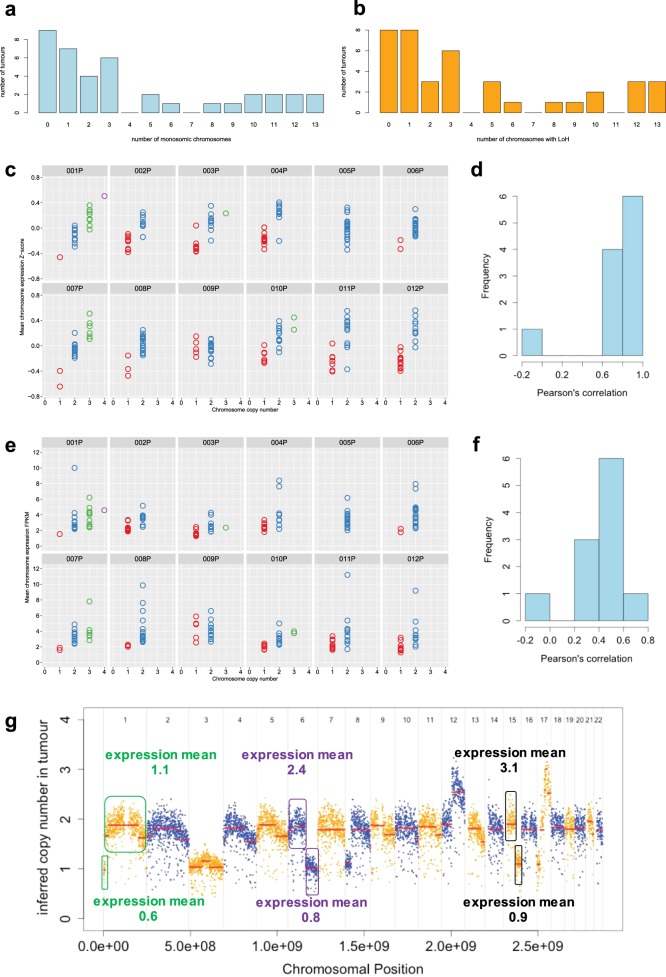
pNET aneuploidy is extensive but varies between tumors. **a** Histogram shows the number of monosomic chromosomes (i.e., whole chromosome LoH with CN = 1) in individual primary tumors. **b** Histogram shows the number of chromosomes with LoH (irrespective of CN) in individual primary tumors. **c–f** Graphs compare whole chromosomal CN (*x*-axis) to mean chromosomal RNA expression based on **c** microarray data or **e** RNAseq data (*y*-axis). Each panel represents a different tumor and each circle represents a different chromosome in that tumor. Histogram of Pearson correlation between CN and **d** microarray RNA expression or **f** RNAseq RNA expression in each tumor. **g** CN across the genome of the tumor 009P that had negative CN-expression correlation (seen at left of histograms in **b** and **d**). This intra-chromosomal analysis confirms the association between CN and RNA expression seen at whole chromosome level in the other pNETs. Chromosomal segments with specific CN aberrations are shown in colored boxes, with mean RNA expression within each of these segments based on fragments per kilobase of transcript per Million mapped reads (FPKM) shown

Primary pNETs are frequently aneuploid (Fig. 2), with 77% (30/39) having ≥1 monosomic chromosome (Fig. 3a), 79% (31/39) having LoH of ≥1 chromosome (leading to loss of one allele of genes on the affected chromosomes, Fig. 3b) and 26% (10/39) having LoH of ≥8 chromosomes (Fig. 3b). In the aneuploid pNETs, whole chromosome CN was associated with whole chromosome mean RNA expression, shown for 12 pNETs (001P-012P) that had been analyzed by both expression microarray (Fig. 3c, d) and RNAseq (Fig. 3e, f). Although one pNET (009P) appeared to have a low negative association between whole chromosome CN and whole chromosome mean RNA expression, this was due to segmental intra-chromosomal CN variation (Fig. 3g), explaining the low correlation observed at whole chromosome level and confirming the observed association between CN and RNA expression. Similar relationships were seen between whole chromosome CN and whole chromosome mean RNA expression for all 32 pNETs without intra-chromosomal CN variation (Supplementary Fig. S3a and b). Importantly, this CN-RNA expression relationship was evident for chromosome 11 (Supplementary Fig. S3c), with significantly reduced mean expression of chromosome 11 genes (Mann–Whitney *U* test *P* ≤ 0.01) observed in those pNETs with whole chromosome 11 loss (Groups 1 and 2 in Fig. 2) compared to those pNETs with whole chromosome 11 intact.

### Patterns of stereotyped aneuploidy and gene expression are associated with clinical behavior

Previous work by Nagano et al.^7^ and Scarpa et al.^9^ identified patterns of chromosomal changes shared between subsets of pNETs. In the pNETs of this study, we found clear CN groups similar to those previously identified and noted strong correlations between CN group and clinical behavior. Three distinct pNET groups based on CN change emerged (annotated above Fig. 2). In 10 pNETs (labeled Group 1 in Fig. 2), there was a recurrent pattern of LoH affecting the same 10 chromosomes (1, 2, 3, 6, 8, 10, 11, 16, 21, 22), which has been previously noted.^7,9^ This idiosyncratic pattern of aneuploidy occurred in the context of somatic *MEN1* mutation in nine of 10 tumors, an *ATRX* or *DAXX* variant was present in five, with additional *PTEN* or *TP53* mutations present in four. RNA expression analysis showed that *MGMT* (encoding DNA repair protein O-6-Methylguanine-DNA Methyltransferase) was generally expressed at lower levels in these 10 tumors than in other tumors (*t*-test *P* < 0.01). Microarray methylation analysis showed *MGMT* gene promoter methylation was relatively consistent across pNETs with no significant correlation to expression (data not shown). Therefore, differential *MGMT* gene methylation, which has been described in other tumor types,^24^ is unlikely to be the dominant mechanism causing lower *MGMT* RNA expression in this group of pNETs. Instead, one copy of chromosome 10 (the location of *MGMT*) was lost in all 10 tumors in this cluster, suggesting haploinsufficiency as a more likely mechanism for reduced *MGMT* expression (Fig. 2).

Tumors in Group 1 had generally less favorable outcomes; four of the 10 tumors in this group had metastasized, this group contained the only three patients who progressed during the study, and all but one tumor had lymphovascular invasion (LVI) on pathological examination. In contrast, tumors in Group 2 (Fig. 2) were characterized by *MEN1* mutation and chromosome 11 LoH but no recurrent LoH of 10 chromosomes. This group had relatively favorable pathological and clinical outcomes; all had low expression of proliferation-associated RNAs, all but one of the 16 tumors in this group were low grade (Ki67 ≤ 2%), only three had LVI and none metastasized. Several patients in this group had a clinical history generally associated with inherited cancer predisposition (multiple cancers, multifocal pNETs, and age ≤40 years). Ten of the 14 tumors in this group for which RNA expression data were available had detectable *GCG* (glucagon) expression. Group 3 (Fig. 2) was characterized by a lack of *MEN1* gene mutation, contained tumors with variable patterns of aneuploidy (ranging from none to extensive) and variable clinical outcomes. We are unable to report on progression-free survival due to unavailability of time to progression data. However, proxies for disease severity such as grade and the proportion of patients with metastases were significantly associated with CN group (Fisher’s Exact test *P* = 0.017 and 0.018, respectively).

### pNETs have few somatic driver mutations or structural genomic lesions

**Fig. 4:**
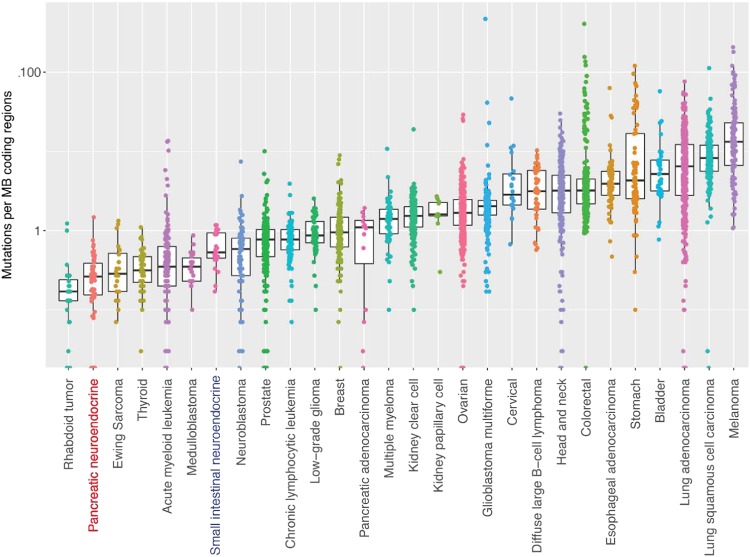
Number of mutations in pNETs. pNETs have relatively low somatic mutation frequency compared to other tumor types; box plots show the coding region mutation rate of pNETs compared to the coding region mutation rates described by Lawrence et al.^25^ in other tumors analyzed by WES or WGS

pNETs have very few detectable somatic variants (Fig. 1, Supplementary Table S5) compared to other tumor types^25^ and only one pNET in this study had more than one variant detected per MB of exons (Fig. 4). Neither large-scale structural variants nor genome duplication were detectable in the data available. However, bi-allelic *MEN1* inactivation was common; 81% of tumors with somatic chromosome 11 LoH had a putative pathogenic variant in the remaining *MEN1* allele (Fig. 2). These variants were distributed across the entire *MEN1* coding region (Supplementary Fig. S2c). Analysis of *MEN1* expression showed that nonsense and frameshift variants were associated with reduced *MEN1* RNA abundance (Supplementary Fig. S2d). This suggests that processes such as nonsense-mediated decay may contribute to reduced abundance of MENIN protein in *MEN1* mutant tumors, in addition to the pathogenic changes introduced by these mutations altering MENIN protein structure and function.

Methylation analysis showed no clear correlation between *MEN1* gene methylation and RNA expression in 15 tumors tested, suggesting that methylation is not an important regulator of *MEN1* expression in pNETs.

MutSig analysis^25^ identified *MEN1* as the only statistically significant cancer driver gene across this pNET cohort, although previously described variants in a small number of other tumor suppressor genes were seen in multiple tumors (Figs. 1 and 2) including *ATRX*, *DAXX*, *PTEN, YY1*, and *VHL*. Private variants in 31 other genes were detected in single patients, some of which were clinically interesting as previously described predictive biomarkers for specific therapies, including bi-allelic inactivation of *MSH2* and mono-allelic variants in genes such as *RET, JAK2, FGFR3*, and *BRCA2* (Fig. 1). Although these variants were classified as functionally significant using combinations of variant effect databases (see Methods) and many are considered clinically actionable using current assessment tools (Supplementary Fig. S4), genomic analyses suggested that most were passenger mutations rather than drivers of tumorigenesis. For example, examination of variants in genes encoding tyrosine kinases that have matching small molecule inhibitors found no corroborating *JAK2* (patient 021), *FGFR3* (patient 024), or *RET* (patient 051) gene expression/pathway changes. However, the mutation pathogenicity of variants in one tumor was corroborated by the RNA expression data—tumor 002P had a somatic frameshift mutation in *PTEN* (a phosphoinositide 3-kinase (PI3K)/AKT/mTOR signaling inhibitor) as well as a non-frameshift deletion and LoH in the *FLCN* gene (encodes the mTOR complex 2 inhibitor folliculin). Microarray analysis of the expression of RNAs downstream of PI3K suggested significant activation of PI3K/AKT/mTOR pathways in this tumor, consistent with the mutations (Supplementary Fig. S5).

### Comparison of primary and metastatic pNETs

Two patients in this case series had both primary and metastatic tumors available for analysis (009 and 053) and another had multiple pancreatic primaries that were resected concurrently (patient 028). Supplementary Fig. S6 compares the multiple tumors for these individuals in terms of mutation (Supplementary Fig. S6a) and CN/gene expression/histology (Supplementary Fig. S6b). The two pancreatic tumor regions of patient 028 were indistinguishable in terms of mutation, CN, histology, and gene expression. The pancreatic primary tumor and nodal metastasis of patient 053 were also identical in terms of genome sequence, genome structure, and expression of the *SST* gene encoding the dominant tumor hormone somatostatin. However, small differences in expression of other hormones between tumor and metastasis were seen.

For individual 009, the primary tumor (009P) and two hepatic metastases (009La and 009Lb; Supplementary Fig. S7) share many genomic features. For example, all three share an *ATRX* nonsense mutation (Supplementary Fig. S7b) and concordantly the longest telomeres of any pNETs are in this case series (Supplementary Fig. S7c-d). The primary and both metastases also share a low overall mutation rate and moderate aneuploidy (Supplementary Fig. S7e-f), moderately high expression of the *MKI67* RNA encoding the proliferative protein marker Ki67 (Supplementary Fig. S7g) and high mean expression of the combined Cybersort gene sets^26^ (as a global marker of immune cell infiltration; Supplementary Fig. S7h). In addition, the primary and metastatic tumors of this individual also shared high expression of the RNA encoding ghrelin (Supplementary Fig. S6b); however, neither metastatic tumor carried over the expression pattern of *GCG* and *SST* RNAs seen in the primary. Although the interpretation of chromosomal losses and gains was complicated by high and variable stromal content in this patient’s tumors, it is clear that the metastases retain the LoH of chromosomes 3 (full), 6 (partial), 8 (partial), and 17 (partial, mixed with amplification) seen in the primary tumor. However, LoH of part of chromosome 11 in the primary tumor was not carried over into the hepatic metastases, yet both metastases gained de novo LoH of parts of chromosomes 1 and 15 (Supplementary Fig. S8a-c). The regional chromosome 17 LoH/amplification seen in all three tumors was associated with concordant changes in gene expression (Supplementary Fig. S8d), as was the partial LoH in chromosome 15 acquired by the metastases but absent in the primary (Supplementary Fig. S8e).

### Pseudohypoxia determines the expression profiles of some pNETs

Tumors from six patients (eight samples) had high expression of a subset of the hypoxia-activated RNAs. These tumors also tended to have more rapid proliferation based on both *MKI67* RNA expression (Supplementary Fig. S9a) and immunostaining (seven of these tumors were grade 2 NETs with Ki67 immunostaining in 3–20% of nuclei). However, further genomic analysis showed that two of the eight tumors had somatic *VHL* variants with LoH, and tumors from other patients had high *VHL* gene methylation associated with significantly low *VHL* RNA expression (Supplementary Fig. S9b). This suggests that in the majority of the pNETs analyzed, tumor hypoxia gene expression profiles are due to pseudohypoxia caused by disrupted VHL function rather than true hypoxia.

### Germline variants may become significant in the context of extensive aneuploidy

**Fig. 5:**
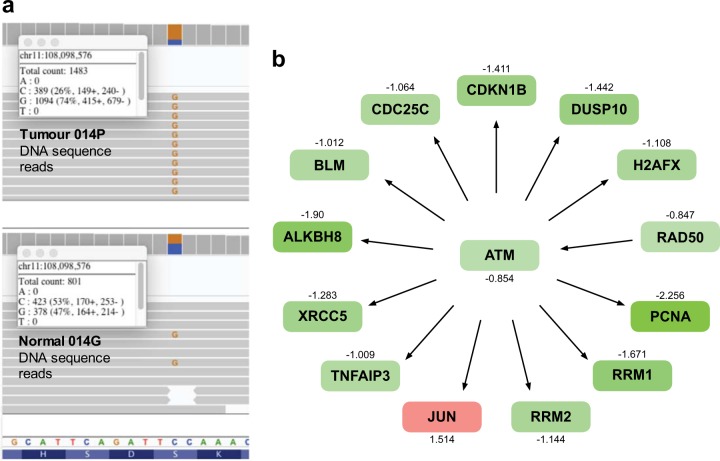
Somatic LoH may expose germline heterozygous variants. **a** Example of a germline heterozygous mutation in the *ATM* locus that becomes unopposed in the tumor 014P due to somatic LoH. **b** IPA analysis indicates that in this tumor, expression of numerous RNAs that are normally upregulated by the activity of the ATM complex is generally reduced, suggesting reduced ATM function. Shades of red and green indicate the degree of up- and downregulation of RNAs, respectively, with *Z*-score expression values shown above or below each node

In our patient series, we were able to exclude with high confidence any functionally relevant germline variants in the following genes previously associated with NETs: *MEN1, RET, TSC1, TCS2, PTEN, NF1, CDKN1B, IPMK, MAX, NF1*, *NTRK1*, *SDHA*, *SDHB*, *SDHC*, *SDHD, MUTYH*, and *VHL*. However, there were 173 germline variants in 66 genes not traditionally associated with NETs that were predicted to disrupt protein function (Supplementary Table S6). The list of genes affected was significantly enriched for genes associated with DNA repair (GO:0006281, *P* = 6 × 10^−9^) using the PANTHER web tool. Eight of these variants appeared to become unopposed when their remaining normal allele was lost by somatic LoH (Supplementary Table S7). These eight variants had ~1:1 ALT:REF allele ratios in germline DNA, and all had somatic LoH in tumor DNA and corresponding tumor ALT:REF allele ratios of ≥1.5. In these tumors, the degree of loss of the remaining normal allele was consistent with the proportion of tumor comprising somatic cells. As an example, tumor 014P had a chr11:108098576_C/G variant in *ATM* with an ALT:REF allele ratio of 0.9 in the germline but 2.8 in the tumor due to LoH (Fig. 5a). This variant has a population frequency of 0.007 in the ExAc database and leads to a p.Ser49Phe amino acid substitution. Although Clinvar indicated this was a variant of uncertain significance, analysis using IPA and GeneSetDB indicated that numerous RNAs with expression dependent on ATM function were downregulated in this tumor (Fig. 5b), consistent with somatic LoH exposing a pathogenic germline variant causing somatic loss of ATM activity.

### Immune, proliferative and hormone expression characteristics of pNETs

The pVAC-Seq neoantigen prediction framework^27^ putatively identified only one tumor (012P) with a mutation capable of generating a potential neoantigen consistent with the patient’s HLA haplotype. This low incidence of predicted neoantigens is not surprising, given the low somatic mutation rate in pNETs (Fig. 4). There was no association between grade and somatic variant frequency, suggesting that tumor grade is not determined by single gene events in pNETs.

RNA expression of pancreatic endocrine hormones was examined to identify sub-clinical functioning (e.g., insulinoma, glucagonoma) and “non-functioning” pNETs. Approximately two-thirds of pNETs had detectable expression of the RNA encoding at least one hormone, despite the absence of symptoms reported clinically by patients. Expression of the RNAs *INS* (encodes Insulin) and *IAPP* (encodes Amylin) appeared correlated, in line with their known co-production by pancreatic islet β cells (Supplementary Fig. S10a). The expression of a set of RNAs not usually noted to be co-expressed in the same islet cells (*GCG*, *PPY*, and *CCK*) also appeared correlated. Tumors expressing *VIP* (encodes Vasoactive Intestinal Peptide) RNA did so exclusively (Supplementary Fig. S10a).

Tumors expressing *GHRL* (encodes ghrelin, produced by ε cells) RNA also did so exclusively, and methylation analysis found that high *GHRL* expression (in the three tumors from patient 009) had low mean methylation of CpG islands in the *GHRL* gene promoter, suggesting that dysregulated methylation may have contributed to *GHRL* expression in this patient (Supplementary Fig. S10b). Differential gene promoter methylation was not associated with RNA expression for any pancreatic endocrine hormones other than ghrelin in the 15 tumors assessed. *INS* RNA expression was only associated with a clinical diagnosis of ‘insulinoma’ (biochemically proven hypoglycemia caused by pNET insulin secretion) in a subset of tumors, and in some tumors there appeared to be *INS* RNA expression without a documented clinical syndrome (Supplementary Fig. S10a).

## Discussion

### Unusual genomic lesions

By combining multiple types of genomic analysis, we have shown that pNETs develop through a range of unusual oncogenic mechanisms. Although more than half of pNETs have biallelic loss of *MEN1*, the overall frequency of somatic SNVs, indels, and structural DNA variants was low, with small numbers of tumors carrying tumor suppressor variants in *ATRX*, *DAXX*, *VHL*, *PTEN*, *YY1*, and *PAX6*. These variants generally accord with those previously observed in pNETs.^9,12,28^

Rather than mutation, it appears that most pNETs are defined by variable and extensive aneuploidy. For example, approximately 80% of pNETs in this series had lost a copy of ≥1 chromosome and a recurrent pattern of aneuploidy was observed in some pNETs, which carried LoH of an identical set of 10 chromosomes, therefore affecting thousands of genes. Somatic haploinsufficiency is a plausible mechanism by which this LoH may contribute to pNET development, supported by the striking association we demonstrate between RNA expression and CN at the level of whole chromosomes and chromosome segments. Given the large number of genes affected on these 10 chromosomes (≥9500), it is difficult to identify gene sets or pathways significantly enriched above what could occur by chance. Nevertheless, a range of tumor suppressor genes are now thought to drive tumor development through haploinsufficiency rather than by simple mutation^29^ and aneuploidy can disrupt entire signaling pathways, especially those that depend on precise stoichiometry of protein subunits.^30^ It is possible that development of some pNETs may be driven predominantly by aneuploidy, analogous to chromosome 5q-deleted myelodysplastic syndrome in which haploinsufficiency without specific mutation appears to drive the neoplasia.^31^ Understanding the origin, selection in tumor populations and clinical consequences of recurrent aneuploidy, such as we see here, remains a key challenge for modern cancer biology.

Haploinsufficiency is a tenable direct cause for the low *MGMT* RNA expression in pNETs with the recurrent pattern of 10-chromosome loss, since heterozygous *MGMT*+/− mouse tissues have significantly reduced O-6-Methylguanine-DNA Methyltransferase activity.^32^ Since low *MGMT* function is one of the determinants of response to alkylating agents such as temozolomide, pNETs with low *MGMT* expression may potentially respond to temozolomide therapy, and this aneuploid genotype may have contributed to variations in response to temozolomide in previously published series.^33^ We also show an example where somatic LoH renders a pathogenic heterozygous germline variant in *ATM* unopposed, thereby inactivating its downstream tumor suppressor pathways (Fig. 5). Careful inspection of the germline in each patient found no evidence of traditional syndromic NET-associated mutations, or the recently recognized *MUTYH* germline mutations^9^ in our cohort.

Distinct patterns of pancreatic islet hormone expression were seen in the majority of pNETs and may indicate the cell of origin of these tumors. Although RNA expression might not translate into protein expression, high expression of RNAs encoding hormones in most pNETs analyzed suggests that clinicians should be aware of under-diagnosis of subtle secretory syndromes. Interestingly, we also observed one patient with three metastatic tumors with high *GHRL*^34^ RNA expression and promoter hypomethylation. A recent study of 26 insulinomas found mutations, CN changes and focal allelic imbalances in genes significantly enriched for epigenetic regulators.^11^ However, in the 12 clinically defined insulinomas in our pNET cohort, neither somatic variants nor the genes affected by aneuploidy showed significant enrichment for epigenetic factors listed in EpiFactors database.^35^

Few molecular differences were apparent when we compared two pancreatic primary tumors from one individual, or a primary and nodal metastatic tumor from another. However, while comparing a pancreatic primary and two hepatic metastases of another individual revealed no differences in SNVs or indels, there was clear progression of aneuploidy, with concordant changes in gene expression apparently associated with the metastatic event. Nonetheless, even in this case, the primary tumor and metastases shared molecular features including the same *ATRX* nonsense mutation accompanied by long telomeres and high expression of an RNA marker of cellular proliferation. In addition, all tumors from this patient shared high expression of immune cell marker genes, suggesting that the drive for tumor immune responses in this patient may be intrinsic to the tumor cells, unaffected by the tissue niche into which metastasis occurred.

### Distinct genomic landscapes, putative oncogenic mechanisms, and clinical features of two pNET subsets

**Fig. 6:**
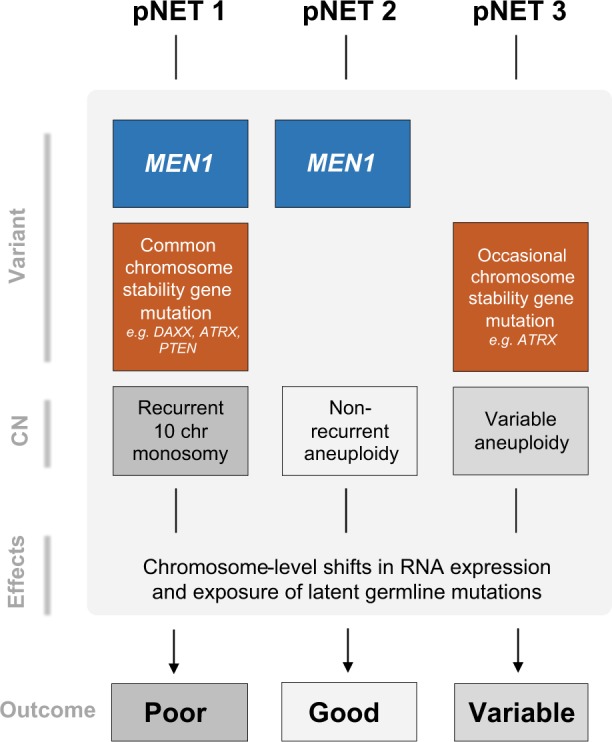
Integrated genomic, pathological, and clinical categorization of pNETs. Genomic features within pNETs identified three groups: Group 1 generally have *MEN1* mutation and chromosome 11 loss, sporadic mutation of genes associated with chromosomal instability, recurrent loss of ten specific chromosomes leading to extensive disruption of gene expression, and reduced *MGMT* expression. These genomic features are strongly associated with high tumor grade and size, LVI, and more frequent metastases. Group 2 have *MEN1* mutation and chromosome 11 loss but no recurrent loss of ten chromosomes. They have universally low tumor grade, size, and LVI, many express *GCG* RNA, and importantly, this group have no metastases. Group 3 are characterized by no *MEN1* mutation, with variable aneuploidy, clinical and pathological features

By combining CN, somatic variant analysis, and expression analysis, we hypothesize distinct oncogenic mechanisms driving two clinically different subsets of pNETs (Fig. 2), summarized in Fig. 6. The first subset, Group 1, are pNETs with *MEN1* mutation coupled with recurrent loss of 10 chromosomes, the cause of which remains unclear. This subset generally had unfavorable grade 2 and 3 histology, all but one patient had LVI and four of the 10 tumors in this group metastasized, and *MGMT* loss through apparent haploinsufficiency may favor the use of temozolomide. The second subset, Group 2, contained pNETs with *MEN1* mutation and chromosome 11 LoH but few other changes in chromosomal CN—none of this group ever went on to metastasize and all but one had favorable low grade histology (Ki67≤2%). In addition, all of this second subset had low expression of proliferation-associated RNAs, only three of the 16 tumors in this subset had LVI and most expressed the RNA encoding glucagon. In this subset, the decision to leave tumors un-resected could be considered in the setting of a clinical trial, thus avoiding the complications and long-term morbidity of surgery for these patients.

## Conclusion

Aneuploidy appears fundamental for pNET tumorigenesis, possibly by altering gene expression on a global scale and exposing pathogenic germline variants, leading to signaling pathway dysregulation. Simple precision oncology paradigms that match drugs to single gene changes will have low utility in tumors with few mutations such as pNETs. However, the strong genotype–phenotype correlation described here, between two pathognomonic pNET LoH patterns and prognosis, is potentially valuable for clinical decisions affecting approximately two-thirds of pNET patients.

## Methods

### Tumor sample collection and processing

Surgically resected, fresh frozen, and FFPE specimens were collected from the Cancer Society Tissue Bank, University of Otago, NZ, and Auckland Region Hospitals under New Zealand Health and Disability Ethics committee approvals 13/NTA/69 and 13/NTB/173 (for further information on sample handling, see Supplementary Methods). This study used both fresh and archival tumor tissue and was hence conducted under two separate ethical approvals from the Health and Disability Ethics Committees of New Zealand. Written informed consent was obtained from all participants from whom fresh tissue was collected. For archival tissue, to avoid potential ascertainment bias when the donors of more aggressive tumors may have died, due to the relative rarity of this tumor type, and with support from NZ’s patient advocacy group for this tumor type, the need for individual informed consent was waived by the Health and Disability Ethics Committees of New Zealand on the condition that these samples were anonymized prior to any analysis being conducted. Matched blood and normal adjacent tissue, where possible >20 mm distant, were used as germline controls for the fresh frozen and FFPE cases, respectively. Frozen tissues were processed to isolate genomic DNA (Macheray Nagel; Nucleospin Tissue kit; #740952) and total RNA (Ambion miRvana RNA isolation kit; Thermo Fisher Scientific; AM1560). FFPE tissues were macro-dissected on slides to maximize tumor cellularity and gDNA and RNA isolated by QIAamp DNA FFPE kit (Qiagen; #56404) or Ambion RecoverAll kit (Thermo Fisher Scientific; AM1975). Whole blood/buffy coat and dried FTA blood spots (6 × 3 mm punches) were extracted using QIAamp DNA mini kits (#51102, #51304). All isolation kits were used as per the manufacturer’s instructions. Nucleic acid quality and quantity were determined by Agilent Tapestation and Qubit Fluorometry, respectively.

### RNA analysis

All microarray hybridization and sequencing machine runs for RNAseq were performed as a service by New Zealand Genomics Ltd. For RNAseq, 100 ng RNA from tumors 001P-012P were used as templates to prepare separate total and mRNA libraries using TruSeq Stranded Total RNA Sample Prep kit with Ribo-Zero gold and TruSeq Stranded mRNA Sample Prep kits, respectively. They were sequenced as a multiplex of six samples per HiSeq lane using V3 chemistry, 2×100PE reads. The total RNA and mRNA sequencing reads were trimmed using cutadapt^36^ v1.9.1 to remove leftover adapters, any reads with Phred score of <30, and read pairs where either read was <50 bp after trimming. Reads were aligned using Bowtie 2^37^ with recommended settings. Aligned total RNA reads and mRNA reads were merged using Picard MergeSamFiles before gene and transcripts abundance quantified using RSEM.^38^ Fusion genes were searched for using TopHat-Fusion.^39^

For microarray expression analysis, Affymetrix PrimeView Human Gene Expression arrays were used (perfect-match-only microarrays with ~530,000 probes covering ~36,000 transcripts). A total of 100 ng RNA was labeled using the Affymetrix SensationPlus FFPE method according to manufacturer instructions, before hybridization to the gene chips, washing and scanning. QC was performed using Affymetrix Expression Console and in-house R scripts to visualize probe signal distributions relative to control signals. Data for one tumor (015P) were discarded due to very low tumor-derived signals relative to spiked-in control signals, and remaining tumor data were quantile normalized in R using RMA,^40^ as implemented in the R ‘affy’ package.^41^ To remove any systematic FFPE-vs.-fresh frozen sample biases, for each probe, the mean signal within all FFPE tumors was subtracted from each individual FFPE tumor’s signal, with an identical adjustment performed within the fresh frozen tumor group. A comparison between the results of RNAseq and microarray analysis revealed relatively high concordance and is shown in Supplementary Fig. S11.

For all visualizations, expression values for each probe set were transformed into *Z*-scores relative to all tumors in the analyzed cohort (by mean centering the data, then expressing the variation above and below the mean on a scale of standard deviation) and all analysis of Probe set differential expression used the LIMMA R package.^42^ Differentially expressed probe sets were tested for enrichment of particular functional categories or pathways using IPA^43^ and GeneSetDB.^44^ Stromal content was estimated from sequence data using ADTEx,^45^ with Immune subtype abundance in the tumors estimated using the Cibersort^26^ and Estimate^46^ methods. To generate Fig. 2, the R Shiny package was used to visualize mRNA expression alongside clinical and pathological information for specific gene sets.

### Methylation analysis

A total of 500 ng of gDNA from each of the 001P-012P, 009La, 009Lb tumors was bisulfite converted as per manufacturer instructions for the EZ DNA methylation kit (Zymo; D5001). Samples were labeled and hybridized as a service by AgResearch Ltd., GenomeNZ section, New Zealand, onto Illumina Infinium Human 450k methylation arrays. Data were visualized and QC performed using the ChAMP package,^47^ which also provided estimates of tumor gene CN. All methylation BeadChips passed QC standards recommended in the ChAMP documentation. Methylation *β* values were subsequently extracted from the idat files with the RnBeads^48^ package in R using the hg19 human genome assembly and mean aggregation of each of: promoters, CpG islands, and genes. Measurements were filtered using the Greedycut algorithm; background was subtracted using the noob method of the methylumi package before signal intensity normalization using the SWAN method of the minfi package.^49^ mRNA expression data (microarray) and gene methylation data were linked through Ensemble gene ID of the respective platform annotation files. A local MySQL database was generated and queried through statistical filters (significance of correlation and level of expression) to identify significantly anti-correlated/correlated methylation and mRNA expression, using the RSQLite package.

### DNA sequencing and data analysis

WGS libraries were generated for tumors 001P-012P using a Rubicon ThruPLEX-FD kit with 3–50 ng of input DNA. For shallow WGS each WGS library was run 1 sample per lane of HiSeq (but split over multiple lanes) with V3 Chemistry 2×100PE reads. WES enrichment was performed using the Agilent SureSelect V5 + UTR systems on the above libraries and run in a multiplex of 3 per lane as per the WGS analysis. For targeted sequencing, the NimbleGen SeqCap EZ comprehensive cancer panel was used (Roche; #4000007080—a ~4 Mb design that targets 578 cancer-related genes). An additional custom SeqCap EZ choice panel (Roche NimbleGen 06266282001) covering 59 additional genes with a capture space of 354 Kb was also designed (Custom NET panel) (Supplementary Table S3). This was completed according to manufacturer instructions, and as further described in the Supplementary Methods.

Sequencing reads were quality trimmed using cutadapt v1.9.1 to remove left over Illumina specific adapters and any reads with Phred score of <30. Read pairs were removed if either read had an after trimming length of <50 bp. Reads were aligned to UCSC hg19 reference genome using BWA-mem^50^ with default settings and duplicated reads then removed using Picard v2.1.0 MarkDuplicates (http://broadinstitute.github.io/picard/). Aligned reads with minimum mapping quality of 1 were selected using Samtools.^51^ Due to the high sequence depth achieved by target capture, maximum depths were set to 9000, the Samtools per-Base Alignment Quality calculation was removed, and tumor purity was set to 50%. Finally, the strand filter was removed as it is not applicable to target-captured data. The R SomaticSignatures package^52^ was used to identify and plot mutational signatures using non-NMF (Supplementary Fig. S2), with putative origins of mutational signatures based on information in Alexandrov et al.^53^

### Variant calling and annotation

Somatic SNVs and indels were primarily detected using the VarScan2^54^ v2.3.7 somatic workflow. Somatic variants were detected in parallel using Strelka^55^ and qSNP^56^ without filtering with default settings—all SNVs and indels described in this paper could be detected using all three methods. Neither Varscan’s germline nor somatic *p*-value filters were used. VCF files were extensively annotated using PERL scripts modified from ANNOVAR (using ANNOVARs ljb26 database) with additional annotations from The Cancer Genome Interpreter (https://www.cancergenomeinterpreter.org). Variants detected in presence of supplementary reads were additionally annotated with a custom flag in the original VCF file using vcf-annotate in VCFtools.^57^ Somatic variants were then filtered in real time, while visualizing the effects of the filtering, using the R VariantAnnotation and R Shiny packages. It used the R DNAcopy package^58^ for circular binary segmentation subroutines and for visualization of segmental CN aberrations and corresponding segmental B-allele frequency changes. Post-calling filtering used the following criteria: Normal tissue and tumor read depth at the site of the mutations ≥50, ≥10 tumor sequences showing the mutation, the site of mutation is not within the Encode Dac Mapability black list, and ≤2 reads corresponding to the mutation were found in the germline sample. All somatic variants that passed these filters were visually validated in IGV. Germline SNVs and indels were detected using the VarScan2 v2.3.7 germline workflow. Mutation plots and lollipop plots in Figs. 1 and 2 and Supplementary Fig. 2c were generated using modifications of the waterfall and lolliplot functions, respectively, of the GenVisR R package.^59^

### Coding region mutation rate analysis

The pNET coding region mutation rate was compared to the rates described by Lawrence et al.^25^ in other tumor types, which had been analyzed by either WGS or WES. Mutation frequencies were calculated in each pNET based on the numbers of coding region mutations found in regions of the genome with ≥50× sequence coverage. Note that pNET mutation frequencies may be overestimated in this analysis since, compared to the WGS or WES analysis used to calculate coding region mutation rates for the other tumor types, the hybridization capture analysis used here is enriched for cancer-associated genes, which may be more likely to carry mutations.

### CN and structural variant analysis

CN analysis was performed using the deep targeted sequencing data for all tumors and separately using the WGS data available for tumors 001P-012P. CN variation was first visualized by counting the number of reads mapped to 3 kb tiles of the hg19 human genome using bedtools multicov, then analyzed using the DNACopy R package. For each 3 kb tile, all raw counts were log2-transformed, normalized using loess splining and log ratios between tumor and normal were calculated, the log ratios were smoothed, segmented (circular binary segmentation), and visualized. B allele frequencies were also analyzed across the tumor genomes to combine with CN information in order to identify the combination of intra-tumoral heterogeneity and unbalanced chromosomal amplification. To do this required identification of germline heterozygous positions, which was based on: 0.4 < proportion of ALT reads in germline <0.6 and probability ≥0.95 of the observed germline sequence reads being sampled from a population of reads where ALT and REF alleles were equally common, calculated using a binomial distribution. Somatic CN aberrations were also analyzed in parallel using the Varscan2 CN pipeline, supplemented by statistical analyses using ADTEx^45^ and Titan,^60^ and for some tumors, CN information from Infinium Methylation BeadChips were analyzed using ChAMP. WGS, WES, and SeqCap aligned BAM files for patients 001–012 were merged using Picard MergeSamFiles before somatic structural variants were analyzed using MANTA,^61^ Delly2,^62^ and GRIDSS^63^ using default settings; in the other tumors, structural variants were analyzed using these three packages from SeqCap data alone.

### Data availability

The data sets generated and analyzed in this study are not freely available out of respect for cultural considerations about genomic data of New Zealand’s Māori people, but the data are available from the European Genome-phenome Archive (https://www.ebi.ac.uk/ega/home; accession number EGAS00001003038) after consideration by a data access committee chaired by the corresponding author.

## Electronic supplementary material

Supplementary Information

Supplementary Tables
