# Supplementary material for: Recurrent loss of heterozygosity correlates with clinical outcome in pancreatic neuroendocrine cancer
Source: NPJ Genom Med. 2018 Jul 20;3:18. doi: 10.1038/s41525-018-0058-3 (PMC6054670; doi:10.1038/s41525-018-0058-3)
Supplement: Supplementary file 1 — Supplementary Information [file 41525_2018_58_MOESM1_ESM.pdf]

SUPPLEMENTARY INFORMATION

Supplementary Methods

Tumor sample collection and processing. Surgically resected, fresh frozen and FFPE specimens were collected from the Cancer Society Tissue Bank, University of Otago, NZ and Auckland Region Hospitals under New Zealand Health and Disability Ethics committee approvals 13/NTA/69 and 13/NTB/173. Under the terms of these ethics approvals, a subset of historical samples were provided under ethical committee approval without the individual consent of patients. The approval to do this was provided after careful and detailed consultation with stakeholders including Māori advisors and patient groups, who provided support for this approach to ensure as many cases as possible of this uncommon cancer were included. As part of the ethics application, the researchers committed to cut the link to patient identifying information before the genomic analysis was conducted, so that all unconsented samples were, from that point, anonymous. This, naturally, limits the amount of follow up that can be done on these patients, with follow up only possible until the point at which the samples were anonymized.

SeqCap EZ Comprehensive Cancer targeted hybridization Capture. Library construction was performed using a maximum of 250ng of gDNA sheared by sonication (Covaris M220) to 200bp as per the manufacturer's instructions. End-repair was then carried out, followed by dA addition and ligation of indexed Illumina sequencing adaptors (SeqCap Adapter Kit A (07 141 530 0010) and SeqCap Adapter Kit B (07 141 548 001)) using the KAPA Library Prep Kit (07 137 974 001). Where scarce input gDNA was available, e.g. for FTA blood spots, library preparation was performed using 5-10ng of gDNA. This was done according to SeqCap EZ Library SR User's Guide 4.2 with modifications: (i) reduced adapter concentration used during adapter ligation in Chapter 3 step 5; (ii) two post ligation clean-ups were used as an alternative to Dual-SPRI® size selection as described in the KAPA Library Preparation Kit Illumina Technical Data Sheet KR0935-v2.14; (iii) ten cycles of SeqCap EZ Pre-Capture LM-PCR were performed as standard library amplification. Samples that were difficult to amplify and failed to produce a library of sufficient quality were later prepared using thirteen cycles of SeqCap EZ Pre-capture LM-PCR. The resulting amplified sample libraries were enriched by hybridization to the SeqCap EZ Designs libraries. One microgram of multiplex DNA sample library pool was used in each hybridization, incubated 47°C for 72 hours, and single stranded capture libraries were recovered and PCR amplified. Following this post-capture PCR (10 cycles) library clean-up was performed using AMPure XP beads (Agencourt) to remove residual primer dimers and the resulting libraries analyzed for quality using an Agilent Bioanalyser High Sensitivity DNA analysis Chip (5067-4626). All hybridization capture libraries were sequenced using an Illumina HiSeq 2500 system with TruSeq SBS Kit v4 - HS (200-cycles) reagents (Illumina), to generate 125bp paired-end reads. The samples were demultiplexed using the CASAVA version 1.8.2 software provided by Illumina. Due to space constraints, one custom NET panel multiplex library consisting of seven samples was sequenced on an Illumina MiSeq to generate 150bp paired end reads.

Sample numbers between analyses. In some cases, DNA extracted from FFPE-processed tumors was not of sufficient quality for all tumors to use for certain analyses. Consequently, sequence data quality was adequate for mutational analysis (identification of SNVs and indels) in 42 tumors (Figure 1 contains these 42 tumors) but adequate for copy number analysis in only 39 tumors. Therefore, Figures 2 and 3 both represent only 39 tumors.

Immunohistochemistry. Five micrometer sections of FFPE tissues were stained by immunohistochemistry (IHC) by diagnostic laboratories in Christchurch and Anatomic Pathology services, Auckland. Immunostains were performed using a Roche Ventana Benchmark Ultra for IHC (Ki-67, DAKO Clone MIB1; Chromogranin A, DAKO Clone DAK-A3; Synaptophysin, Leica NCL-L-Synap-299 Clone 27G12; Trypsin, Santa Cruz Clone D-1; Beta Catenin, Cell Marque Clone 14; CK19, Cell Marque Clones A53 - B/A2.26; panCK, DAKO Clone MNF116; CD45/LCA, Dako Clone 2B11+PD7/26), a Leica Autostainer XL for Special Stains (ABPAS, Merck Schiffs Reagent; Mucin, Amber Scientific Alcian Blue) or a DAKO Artisan for special stains (Alcoholic Congo Red, DAKO). All tissues were pretreated prior to antibody staining using heat-induced epitope retrieval (HIER) with cell conditioning solution (CC1, Roche). Binding was visualized with the Optiview DAB IHC Detection kit (760-700 Roche).

Sanger sequencing. PCR primer pairs (250-500bp) were designed to amplify individual mutations of interest (Results in Table S4). Primer sequences and locations are available on request from the Authors. 10ng of gDNA was used as template for 33 cycles of PCR and the mutation amplified using the KAPA HiFi PCR enzyme (#KK2501) as per manufacturer's instructions and purified using the Qiagen QIAquick purification kits (#28104, #28704) and Sanger sequenced with BIGDYE terminator. DMSO was used during sequencing where the PCR product was >50% GC rich.

Confirmation of *MEN1* mutation in patient 026. 10ng of FFPE gDNA was amplified on a QiaSeq Targeted custom DNA panel (Qiagen Prod. No. 333525). The panel contained 57 amplicons, contact the Authors for design details. The resulting library was then analyzed on an Ion Torrent Personal Genome Machine as per the manufacturer's instructions.

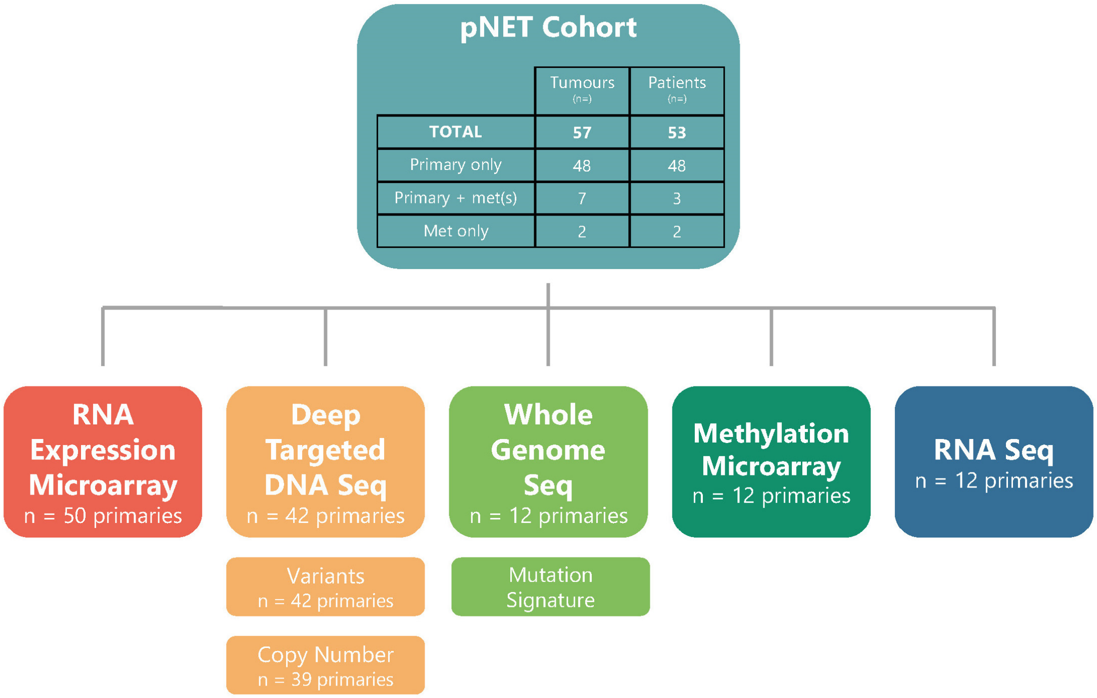

Supplementary Fig. S1. Overall study makeup of pNET cohort. Top box indicates the relationship between primary and metastatic (met) tumors in this cohort: in some cases, we were able to access primary tissue alongside metastases, or met only where no primary tissue was available. This cohort represents a near-sequential series of pNETs collected from New Zealand patients. These samples were analyzed using a range of methods indicated in the workflow chart. In some cases, data was not of sufficient quality for all tumors to use for certain analyses, such as for reliably investigating copy number.

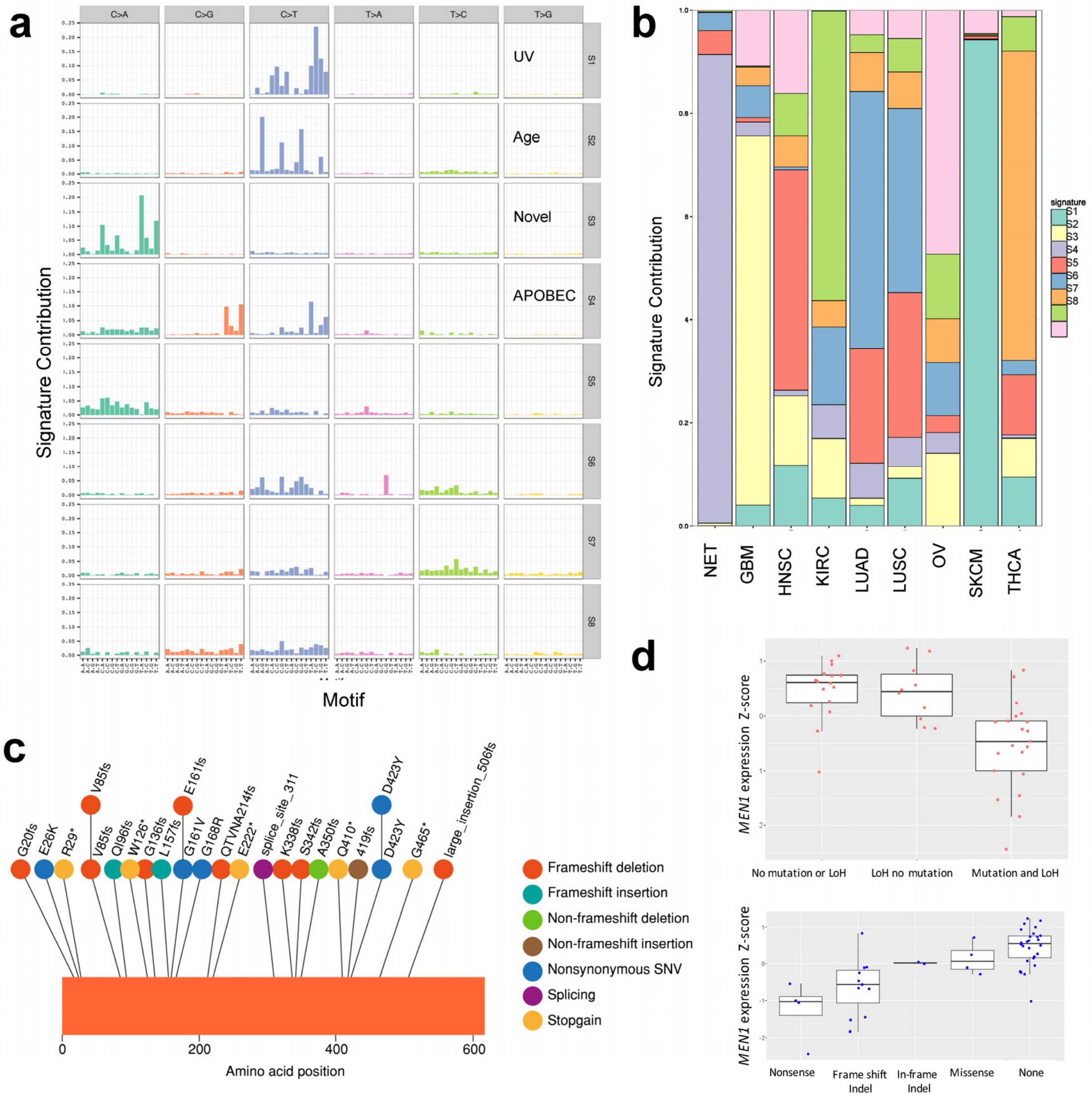

Supplementary Fig. S2. The pNET mutational landscape. (a) Somatic mutations and their immediate contexts were identified in 12 pNETs for which WGS data was available, adding these pNETs to tumors of nine other tumor types using WES data from The Cancer Genome Atlas (TCGA). Eight mutational signatures were identified using NMF. Putative origins of these signatures are suggested based on information in Alexandrov et al (1). **(b)** The contribution of these signatures to the mutational pattern of each tumor type is shown. pNET= pNETs, GBM = Glioblastoma Multiforme, HNSC = Head and Neck Squamous Cell Carcinoma, KIRC = Kidney Renal Clear Cell Carcinoma, LUAD = Lung Adenocarcinoma, LUSC = Lung Squamous Cell Carcinoma, OV = Ovarian serous cystadenocarcinoma, SKCM = Skin Cutaneous Melanoma and THCA = Thyroid Carcinoma. **(c)** Pin plot showing the positions in the MENIN protein isoform encoded by *MEN1* transcript ENST00000377316 that are affected by somatic mutations in the *MEN1* gene. **(d)** Using RNA expression data, the effect of mutation on *MEN1* expression was assessed. Upper panel; *MEN1* gene mutation and LoH (but not LoH alone) is associated with reduced *MEN1* RNA expression. Lower panel; Tumors with nonsense SNVs and frameshift indels have significantly lower (t-test $p \leq 0.01$) *MEN1* RNA expression than tumors with different categories of *MEN1* mutation. (RNA expression data was not available for all tumors shown in Fig. 1).

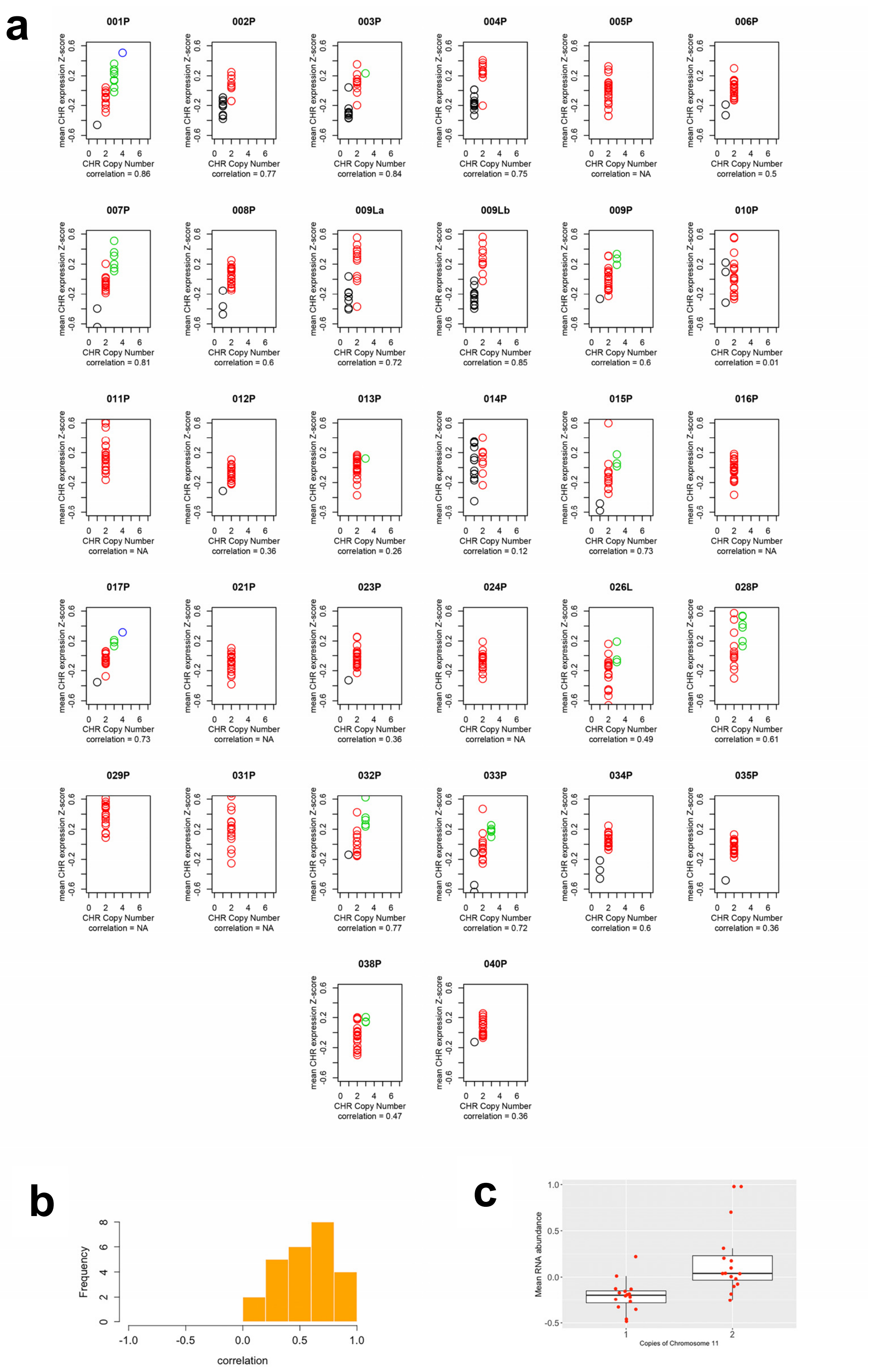

Supplementary Fig. S3. Correlation between pNET chromosomal copy number and RNA abundance. (a) Graphs compare whole chromosomal CN (x-axis) to mean chromosomal RNA expression based on microarray data (y-axis). Each panel represents a different tumor with each circle representing a different chromosome in that tumor. Spearman correlation for each tumor between chromosomal CN and mean chromosomal RNA expression is given below each panel. Only the 32 pNETs without intra-chromosomal CN variation were included. **(b)** Histogram of correlation between CN and microarray RNA expression for the 32 tumors. **(c)** Boxplot of the RNA abundance by copy number specifically for Chromosome 11 in the 32 tumors.

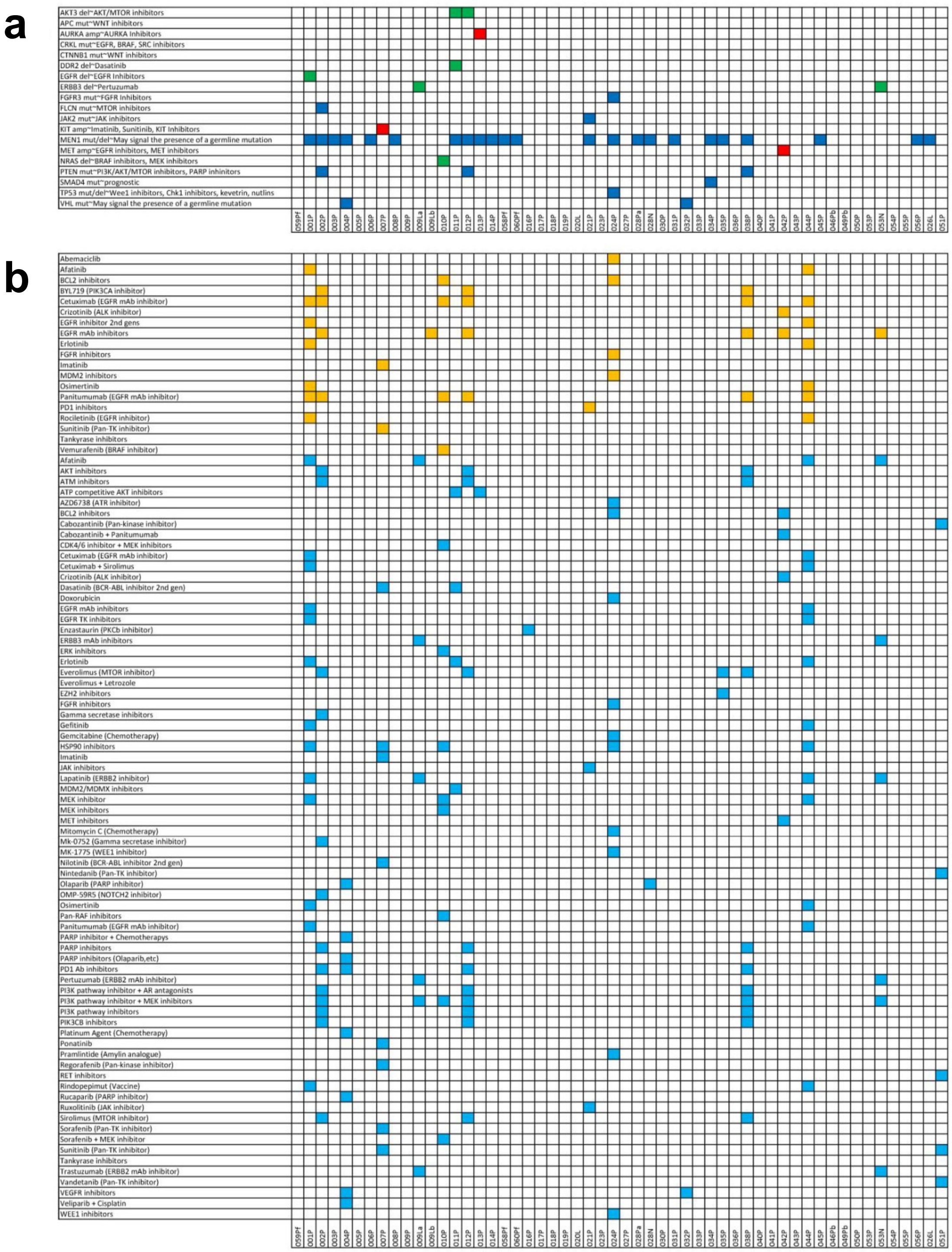

Supplementary Fig. S4. Potential clinical relevance of pNET genomic changes. (a) Putative clinical relevance of somatic DNA mutation and gene expression as identified using the Broad Institute Tumor Alterations Relevant for Genomics-driven Therapy database (Target, v3_02142015). pNETs are shown in columns and genes in rows. Blue boxes indicate potentially relevant somatic mutations. Green and red boxes indicate concordant gene CN change (amplification or deletion) AND gene expression change, where gene indicates expression is up- (red) and down- (green) ≥ 2 SD from the mean of the pNET series. **(b)** The Cancer Genome Interpreter was used to identify, based on both mutations and CN changes, actionable genomic features of the pNET series that may indicate resistance (orange) or response (light blue).

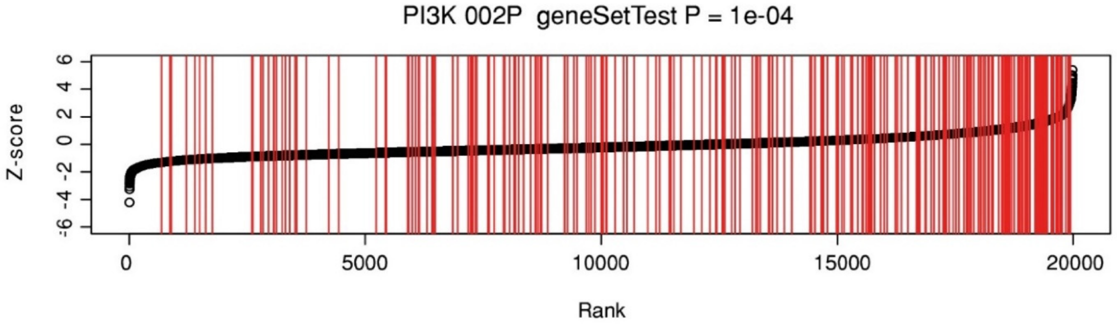

Supplementary Fig. S5. Expression of RNAs downstream of PI3K signaling pathways in a patients with both *PTEN* and *FLCN* mutations. 252 RNAs with expression or transcription directly or indirectly downstream of the PI3K complex were identified using GeneSetDB (2). Expression Z-scores of all RNAs in the transcriptome (y-axis) are plotted against the rank of these Z-scores (x-axis) as black circles (which merge to form a broad black line). Over the top of these circles, the rank in each tumor of the 252 RNAs known to be regulated downstream of the PI3K complex are shown as red vertical lines. Above the graph the result of a geneSetTest (3) is shown ($P < 10^{-4}$), which refers to the hypothesis that the 252 known RNAs downstream of PI3K are more highly ranked in terms of expression Z-scores than other RNAs. IPA (4) upstream pathway activation Z-scores concord with this analysis, for the PI3K and AKT complexes of 4.7 and 3.3, respectively.

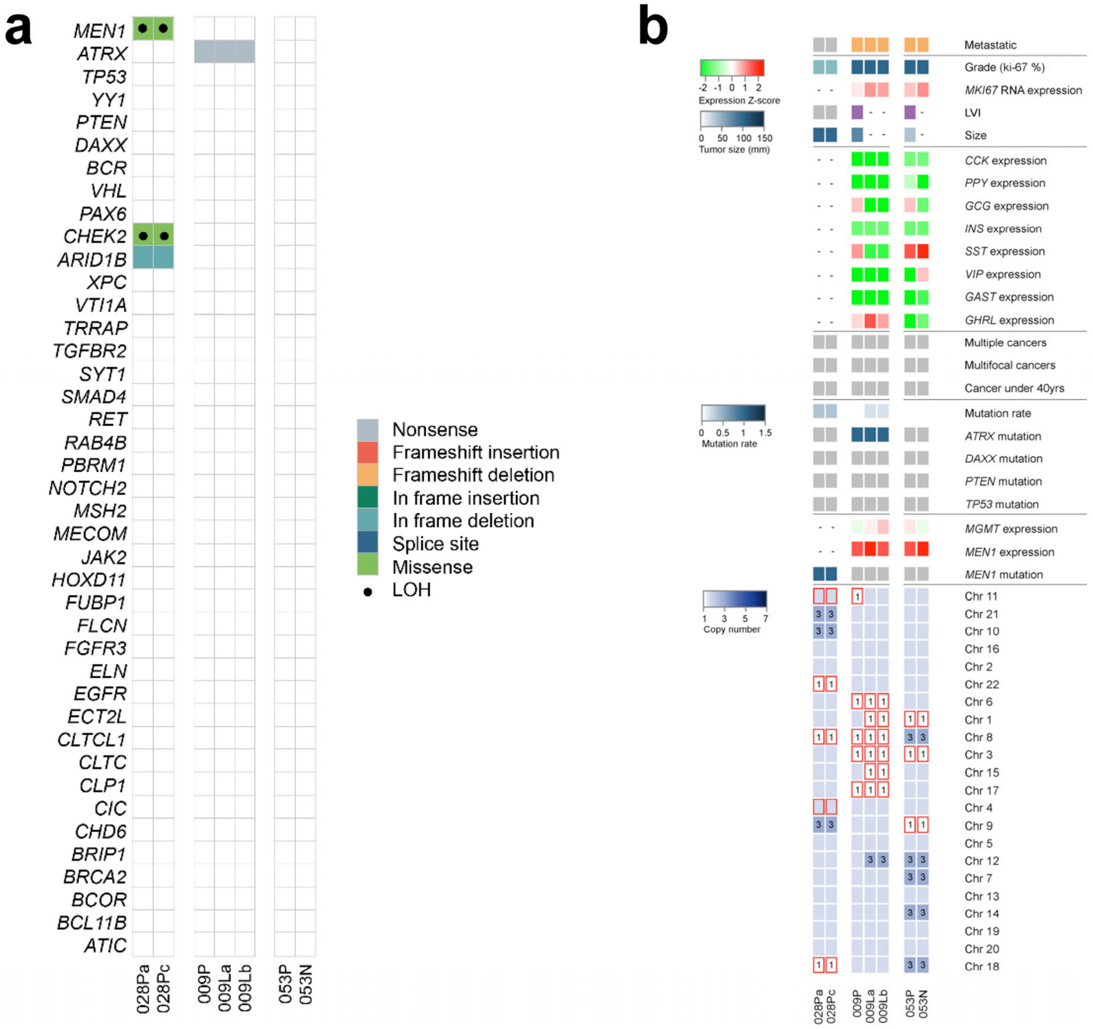

Supplementary Fig. S6. Comparison between primary and metastatic pNETs. Metastatic tumors from the same patients as three primary tumors shown in Figures 1 and 2 are shown. **(a)** Coding region somatic non-synonymous SNVs/indels, large deletions and intronic mutations within 2bp of splice sites with any putative functional significance are shown, figure layout as in Fig. 1. **(b)** Genomic copy number and allele frequency with pathological features are shown, figure layout as in Fig. 2.

a

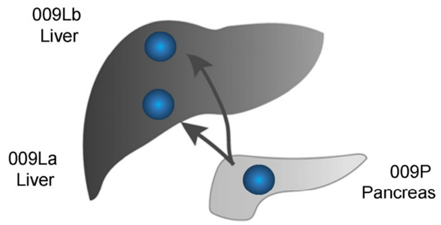

b

ATRX chrX:76813035	C	A	proportion reads supporting variant	proportion tumour cells in sample
normal tissue	1029	1	0	0
009P	1911	405	0.17	~0.3
009La	1061	737	0.41	~0.6
009Lb	1264	537	0.3	~0.5

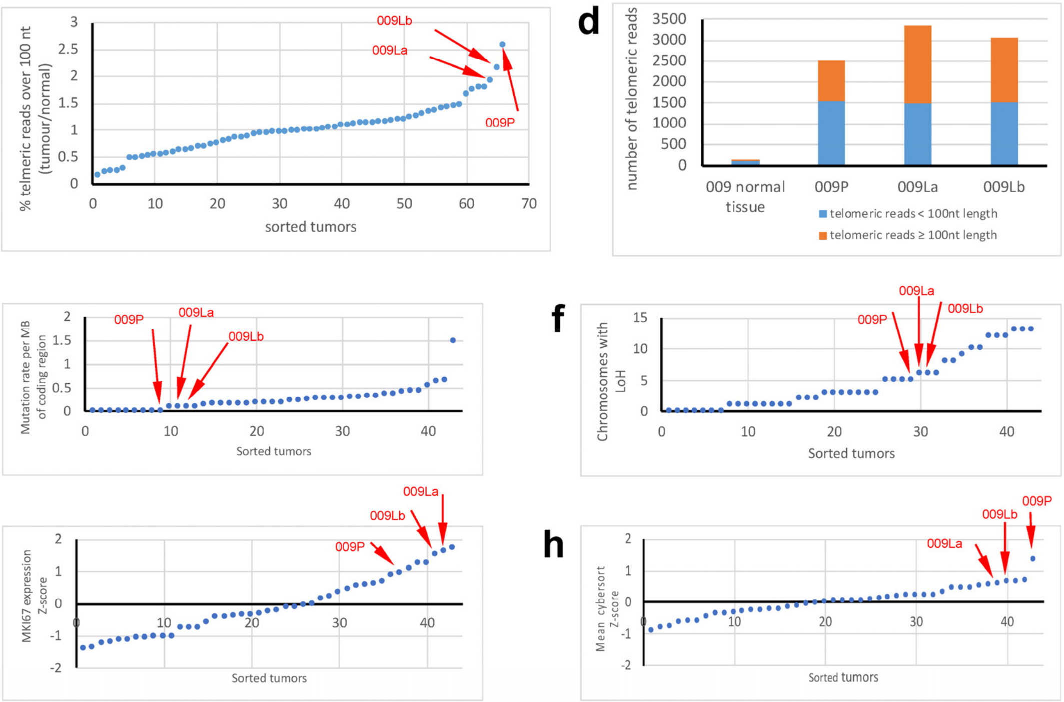

Supplementary Fig. S7. Metastatic tumors of patient 009 share characteristics of the primary. (a) A primary pancreatic tumor (009P) and two separate hepatic metastases (009La and 009Lb) were analyzed. **(b)** A nonsense mutation in ATRX (hg19:chrX:76813035 C>A) causing a premature stop codon in the ATRX protein (p.E2196*) is found in both primary and metastatic tumors. There is no evidence of LoH in the second X chromosome of this female patient, and the frequency of sequence reads sporting the mutation is consistent with a heterozygous variant in all three tumors, given the proportion of tumor cells in the sequenced samples, some of which have levels of somatic cell contamination/infiltration. **(c)** The tumors in the pNET series shown in Figures 1 and 2 are represented as blue dots, with the three tumors of patient 009 labelled to allow comparison with other pNETs, with tumors sorted according to magnitude of the ratio of (% telomere sequence repeats over 100nt in tumor) / (% telomere sequence repeats over 100nt in normal tissue). **(d)** Comparison of the number of telomere sequence reads between normal tissue and the three tumors of patient 009. **(e)** Tumors sorted by mutations per MB of coding region of tumor DNA. **(f)** Tumors sorted by number of chromosomes with LoH. **(g)** Tumors sorted by expression of the MKI67 RNA encoding the proliferative marker protein ki67**. (h)** Tumors sorted by mean expression of all genes in the Cybersort gene sets (as an approximate RNA expression marker of leucocyte infiltration).

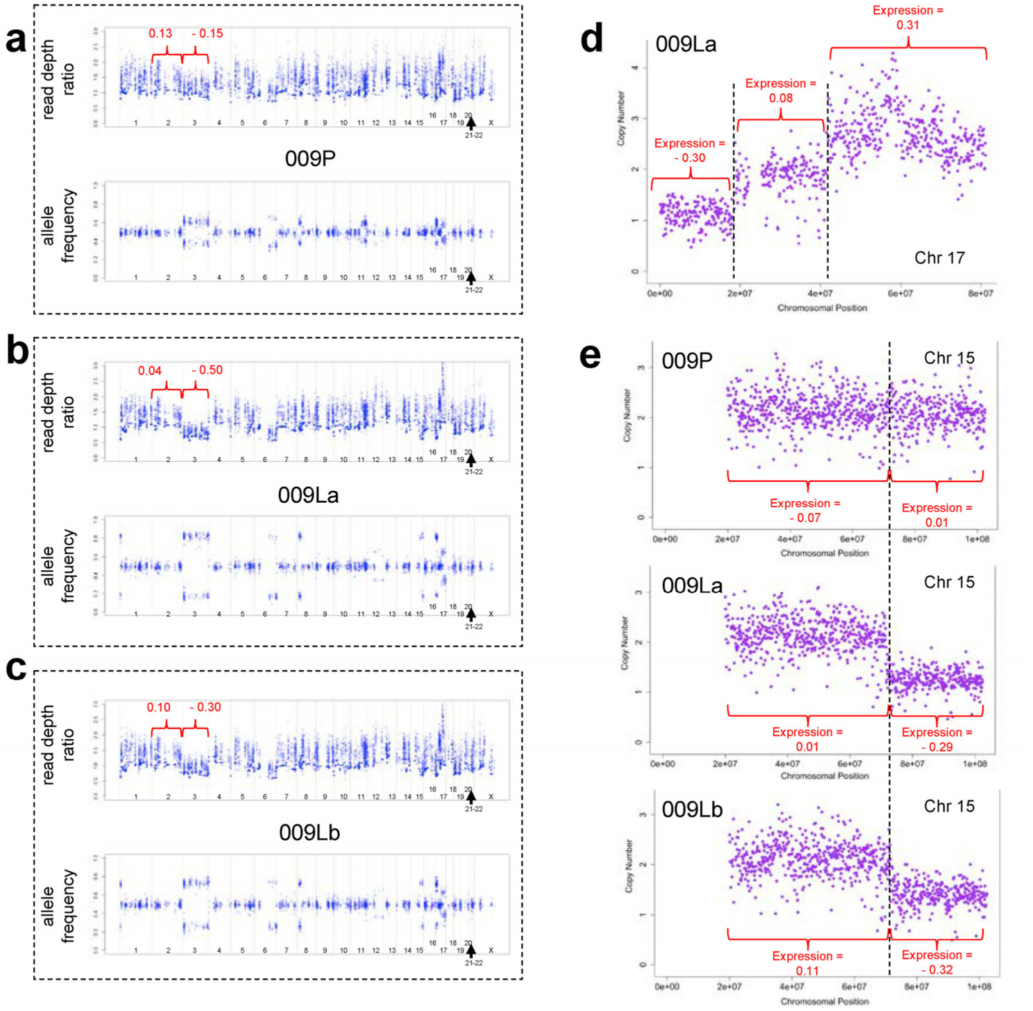

Supplementary Fig. S8. Molecular progression accompanies metastasis in patient 009. (a-c) Primary pancreatic tumor (009P) and two separate hepatic metastases in the same patient (009La and 009Lb) were compared using ADTEx29. For all three tumors, moderated tumor-normal read depth ratio (y-axis in upper panels, proportional to DNA CN) and B-allele frequency (y-axis in lower panels) are shown for each autosome and for chromosome X. The differences between tumors in the degree of B-allele frequency change apparent for chromosomes 3, 6 and 8 can be fully accounted for by different proportions of stromal tissue in the sequenced samples, however other differences appear to be due to genomic progression accompanying metastasis. Both metastatic tumors (009La and 009Lb) have retained LoH of chromosomes 3, 6, 8 and 17 seen in the primary tumor (009P) but also gained de novo LoH of parts of chromosomes 1 and 15. Neither metastatic tumor has carried over the LoH of part of chromosome 11 seen in the primary tumor. All three tumors appear to have lost one copy of chromosome 3, leading to reduced read depth and altered B-allele frequency. Due to less dilution of tumor cells by stromal cells in the two metastatic tumors, this change is visually clearer for 009La and 009Lb tumors than for the primary tumor 009P. The loss of one copy of chromosome 3 DNA appears to have caused reduced RNA abundance; in each tumor, mean gene expression for the entire chromosome 3 (and for the entire chromosome 2 as a control) are shown in red. **(d)** Normalized ratios of tumor-normal DNA read depth in 90kb tiles (y-axis, proportional to DNA CN) are shown across chromosome 17 of metastatic tumor 009La. Three regions of apparently different DNA CN are evident, each of which has different average RNA abundance - the mean abundance of RNAs transcribed from the genes in each region (based on microarray analysis) is shown in red. **(e)** Part of the q arm of chromosome 15, which is normal in the Primary pancreatic tumor (009P), appears to have undergone LoH in the two hepatic metastases (009La and 009Lb). For all three tumors, normalized ratios of tumor-normal DNA read depth in 90kb tiles (y-axis, proportional to DNA CN) are shown across chromosome 15, with mean abundance of RNAs transcribed from the genes in the each region (based on microarray analysis) shown in red.

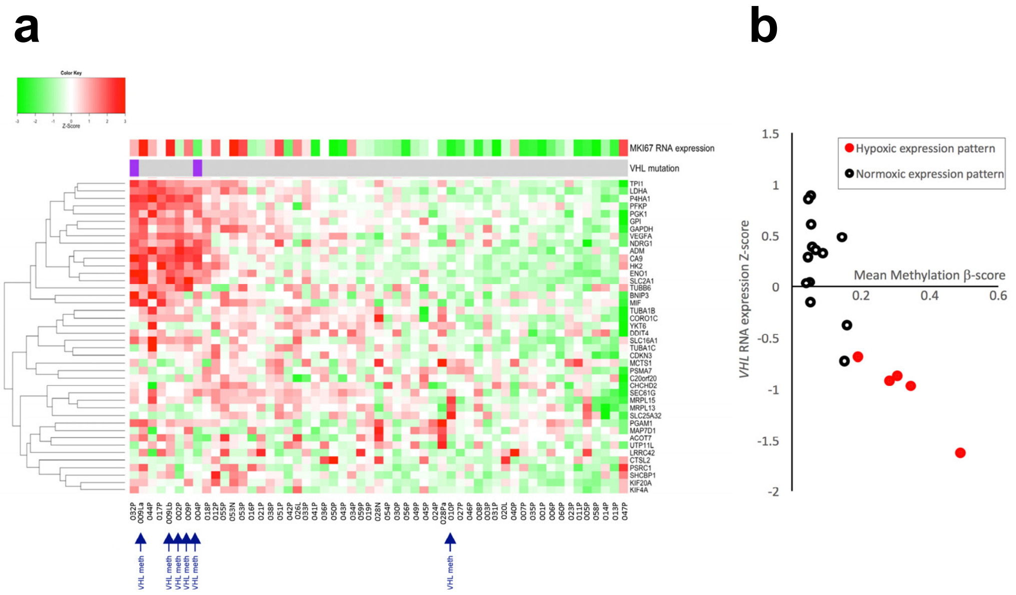

Supplementary Fig. S9. Expression of RNAs associated with hypoxia/pseudohypoxia identifies a subset of pNETs. **(a)** Heatmap shows Z-transformed expression of RNAs associated with hypoxia in cancer (5), sorted according to the first principle component of the RNAs. Although there is no correlated expression for approximately 2/3 of these RNAs, approximately 1/3, which have clustered at the top of the heatmap, have high co-expression in ~ eight of the tumors. Below the heatmap blue arrows indicate six tumors with high *VHL* gene promoter methylation. Above the heatmap is shown: *VHL* gene mutations (indicated in purple) and expression of the *MKI67* RNA (proliferation-associated, green = low, red = high). **(b)** *VHL* gene expression is associated with *VHL* gene promoter methylation. For a series of 18 tumors (all those for which methylation data is available), the mean methylation β-score for the *VHL* gene promoter CpG island (x-axis; assessed using Illumina 450K array analysis) is compared to *VHL* RNA expression Z-score (from microarray analysis, y-axis).

a

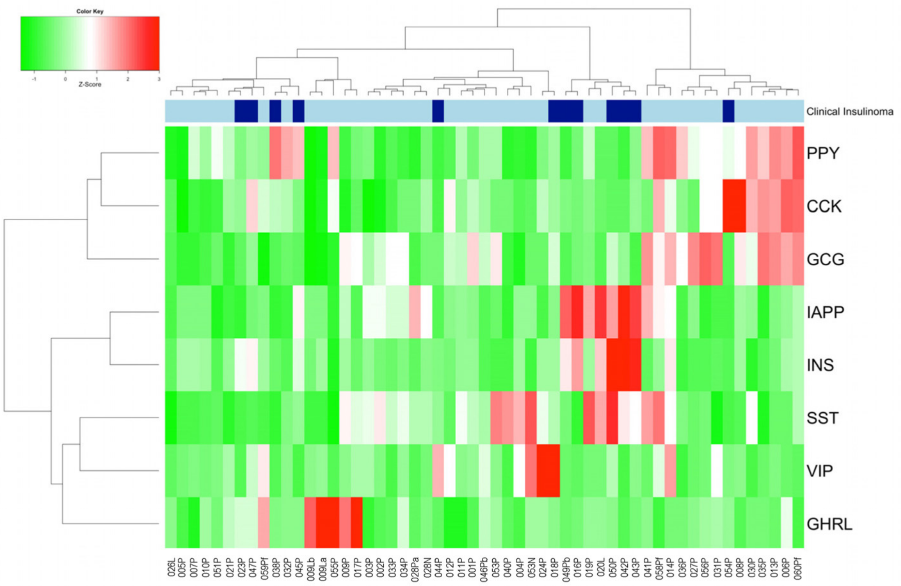

b

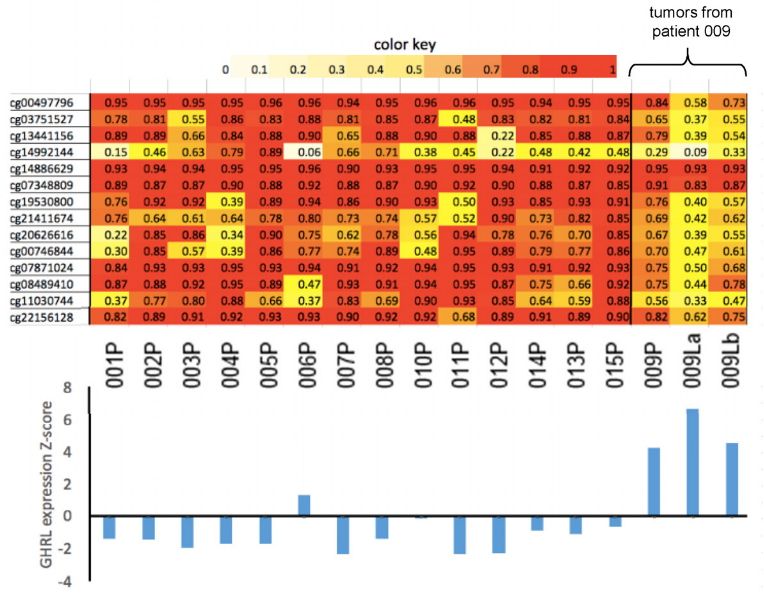

Supplementary Fig. S10. RNA expression of hormone and peptide related genes. (a) Expression of RNAs encoding pancreatic endocrine hormones; heatmap shows Z-transformed expression of RNAs encoding eight pancreatic endocrine hormones clustered using Ward's method and Euclidean distance. **(b)** Ghrelin gene (*GHRL*) expression is associated with *GHRL* locus methylation. For a series of 18 pNETs, Infinium 450K methylation genechip β-scores are shown for CpG probes ordered along the *GHRL* gene (upper panel). Expression of *GHRL* RNA (Z-scores, from microarray analysis) is shown in the lower panel. White boxes indicate missing data.

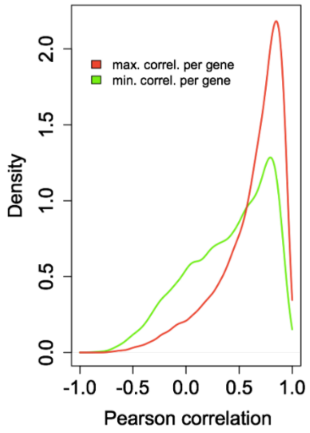

Supplementary Fig. S11. Affymetrix microarray and RNAseq expression data concordance. RNA expression in all tumors was analyzed using Affymetrix Primeview microarrays, and in addition for 12 tumors RNAseq was also used. To evaluate expression data concordance between platforms, normalized mRNAseq FPKM –vs- normalized microarray expression values were compared for each gene; red and green density plots show maximum and minimum Pearson correlation coefficients based on all splice variants of each gene, respectively.

1. Alexandrov LB*, et al.* (2013) Signatures of mutational processes in human cancer. *Nature* 500(7463):415-421.
2. Araki H, Knapp C, Tsai P, & Print C (2012) GeneSetDB: A comprehensive meta-database, statistical and visualisation framework for gene set analysis. *FEBS Open Bio* 2:76-82.
3. Mootha VK*, et al.* (2003) PGC-1α-responsive genes involved in oxidative phosphorylation are coordinately downregulated in human diabetes. *Nature genetics* 34(3):267-273.
4. Kramer A, Green J, Pollard J, Jr., & Tugendreich S (2014) Causal analysis approaches in Ingenuity Pathway Analysis. *Bioinformatics* 30(4):523-530.
5. Buffa F, Harris A, West C, & Miller C (2010) Large meta-analysis of multiple cancers reveals a common, compact and highly prognostic hypoxia metagene. *British journal of cancer* 102(2):428-435.
